# The Effects of Extended Exposure to Supercritical CO_2_ on Select High-Performance Alloys

**DOI:** 10.1021/acsomega.5c11290

**Published:** 2026-06-02

**Authors:** Margarita Ilinich, Taylor Robertson, Dongyi Seo, Kourosh Zanganeh, Holly Dole, Hamid Radfarnia, Henry Saari, Ashkan Beigzadeh

**Affiliations:** † 142123 Natural Resources Canada (NRCan), 1 Haanel Drive, Ottawa, Ontario K1A 0E4, Canada; ‡ 6356 National Research Council of Canada, 1200 Montreal Road, Ottawa, Ontario K1A 0R6, Canada; § 6339 Carleton University, Department of Mechanical and Aerospace Engineering, 1125 Colonel by Drive, Ottawa, Ontario K1S 5B6, Canada

## Abstract

Supercritical carbon dioxide (sCO_2_) Brayton power cycles are being evaluated as alternative to conventional Rankine steam cycle by the power generation industry. sCO_2_ cycles have the capability to be coupled with a variety of heat sources (fossil fuels, solar, nuclear), while providing the potential for increased efficiency, compactness, and lower operating costs. However, the aggressive operating conditions remain a challenge in selecting the appropriate materials of construction. This study investigates the behavior of high-performance alloys, Inconel 740H, Inconel 625, Haynes HR-120, and stainless steel 316L, in long-term exposure to sCO_2_. The materials were exposed to conditions approximating those at the turbine inlet of an indirectly fired sCO_2_ cycle (700 °C and 200 bar) for just over 4800 h. Within the time window examined, the mass gain behavior showed that both Inconel superalloys exhibited an initial parabolic relationship up to 2000 h, while a slight deviation to a more linear relationship was observed for the remainder of the exposure. Between 1500 and 2000 h of exposure, Haynes HR-120 experienced breakaway oxidation behavior, while 316L stainless steel experienced significant spallation. The results of SEM, EDX, and XRD scans showed that Inconel 740H formed the most structurally stable oxide layer composed of a uniform Cr_2_O_3_ outer layer, with underlying discontinuous Al_2_O_3_ oxides. Although Inconel 625 exhibited the lowest mass gain, the presence of heavy elements within its oxide layer is expected to impact the stability of the oxide layer in the long term, whereas the continuous and structurally stable scale formed on 740H indicated superior long-term oxide integrity relative to the other alloys.

## Graphical abstract

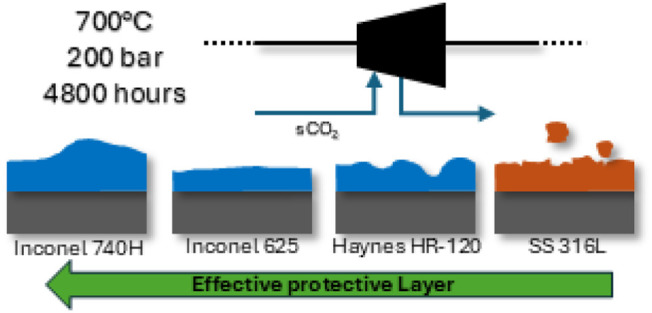

## 1 Introduction

The supercritical carbon dioxide (sCO_2_) Brayton power cycle has been identified as a promising alternative to the conventional steam Rankine cycle in the power-generation industry. Over the years, this technology has accumulated significant interest for applications in fossil, solar, and nuclear power systems due to its high efficiency and compactness, with several collaborative facilities under development by leading research institutions.^1–5^ Indirectly fired sCO_2_ cycles can be coupled with a number of heating sources, which can be a part of fossil fuel, solar, or nuclear heating loops.^1,4–6^ The cycles can further be used in thermal energy storage systems.^7^ Recent reviews and case studies highlight its potential for commercialization and integration with concentrated solar power (CSP) as well as the feasibility of dry cooling and the associated thermal performance challenges.^8–12^

The unique thermophysical properties of sCO_2_ allow it to behave like a gas while having the density of a liquid at conditions above the critical point (31.0 °C and 7.38 MPa). These properties lead to a higher energy production per degree centigrade, allowing sCO_2_ Brayton cycles to operate with higher cycle efficiency and lower operating costs at temperatures above ∼550 °C compared to steam cycles at equivalent temperatures. While these technologies have many advantages, there remain several engineering challenges that need to be overcome before the successful commercial deployment of sCO_2_ power facilities. One of these challenges is the material selection for the construction of the hot sections of the cycle; the peak operating conditions are upward of 700 °C at 25 MPa for indirectly fired cycles, and 1150 °C at 30 MPa for directly fired cycles.

In the past decade, there has been a significant effort undertaken to research the capabilities of various high-performance alloys such as superalloys and austenitic steels for use in sCO_2_ power cycles.^13–15^ Superalloys, such as Inconel 625 and Inconel 740H, are among the leading material choices for high-temperature piping sections of sCO_2_ cycles due to their high-temperature rating and extensive application in steam cycles. Austenitic steels, such as 316/316L stainless steel, are common materials of construction that are used primarily in lower-temperature process areas due to their relatively low cost but high pressure/temperature rating and corrosion resistance. Recent reviews further emphasize that corrosion and carburization behaviors in sCO_2_ depend strongly on the alloy chemistry, impurity levels, and microstructural stability. Xu et al.^14^ highlight that impurity-driven oxidation and carburization remain among the largest uncertainties for high-temperature sCO_2_ Brayton systems, particularly for Cr- and Fe-rich alloys. Kang et al.^15^ similarly conclude that although Ni-based alloys generally outperform stainless steels in sCO_2_, long-term exposure data, especially beyond 1500 h, remain insufficient for reliable lifetime prediction.

The oxidation rate of alloys in sCO_2_ increases with temperature, in accordance with the temperature-dependent diffusion equation for oxygen and metal ion movement. However, there is no clear consensus on whether pressure has a significant impact on the oxidation behavior. Recent studies^16–19^ suggest that as long as CO_2_ is kept in the supercritical state (i.e., *T* > 31.0 °C and *P* > 7.38 MPa), pressure does not have a substantial impact on the oxide scale formation. However, Pint et al.^20^ argued that this behavior is only applicable at extended exposures (<1500 h). Pint et al.^20^ conducted autoclave experiments at low flow rates (2–3 mL/min) to compare the oxidation behavior of alloy 625 at 0.1 MPa in a CO_2_ environment and 30 MPa in an sCO_2_ environment up to 8000 h. The study showed that the initial oxidation (<1500 h) at 30 MPa produced a thicker oxide scale than that at 0.1 MPa, however, with the extended exposure time, the thickness of the oxide scale at both pressures was roughly the same. Liang et al.^21^ and Olivares et al.^22^ showed that while the oxide formation is not greatly impacted by sCO_2_ pressure, the rate of carbon penetration into the material is highly pressure dependent. Pint et al.^20^ and Olivares et al.^22^ found carburization behavior to be of concern in alloys with high Fe content. It is theorized that the primary oxide layer produced in these alloys (Fe_3_O_4_) has a higher carbon permeability and provides more transport channels between grain boundaries to facilitate the diffusion of carbon ions.^23^ Bocher et al.^17^ noted that the presence of high Ni and Cr content contributes to the alloy’s carburization resistance at elevated temperatures. Rouillard et al.^24^ studied the effects of carbon penetration below the oxide layer by flowing laboratory-grade sCO_2_ over the samples inside a 1-L autoclave. The authors noted that the diffusion of carbon into the sample increased the microhardness of the material beneath the oxide scale. Cao et al.^25^ observed an increased oxide layer porosity and spallation as a result of carbon entrapment in the inner oxide layer. Liang et al.^21^ proposed evaluating the depth of corrosion in an sCO_2_ environment by considering the total depth affected by the oxide scale, carburization region, and Cr-depletion region. The author argued that together, these parameters present a more accurate representation of the affected region within the substrate.^21^

Oleksak et al.^26^ compared the performance of Inconel 625 and Inconel 740H in low-pressure CO_2_, sCO_2_, and air in the 700–750 °C range. The study suggests that the oxidation response is very similar in CO_2_-containing environments, showing the development of protective Cr_2_O_3_ oxide in all cases, regardless of pressure or media. The authors suggested this is due to the solid-state diffusion mechanisms that control oxide growth.^26^ Pint et al.^20^ showed that while Cr_2_O_3_ is the dominating oxide in alloy 625, in both CO_2_ and sCO_2_ test environments, the scale formed under supercritical conditions is slightly more dense and contains less voids underneath the oxide layer. Furthermore, when compared against oxidation in air, the oxides appeared to have finer grains in CO_2_ and sCO_2_ environments.^20^ Jiang et al.^27^ showed that oxidation of 740H in air at 1050–1170 °C results in the formation of an internal oxidation zone composed of Al_2_O_3_ and TiO_2_, a continuous and dense middle layer of Cr_2_O_3_ containing precipitates of TiO_2_ and an outer layer of NiCr_2_O_4_ spinel.

Furukawa et al.^28^ studied the oxidation behavior of 316FR stainless steel in an sCO_2_ environment (400–600 °C range) for up to 8000 h. The oxide layer was determined to be relatively thin, but the formation of M_23_C_6_ carbides was highlighted near the surface of the substrate.^28^ Rouillard et al.^24^ tested 316L at 550 °C and 20 MPa to show significant formation of a porous outer Fe_3_O_4_ oxide layer and Fe–Cr-rich spinels underneath in a sCO_2_ environment. The authors concluded that the formation of the Fe_3_O_4_ layer facilitates the inward diffusion of carbon beneath the oxide layer, while the voids caused by the outward diffusion of Fe act as sites for internal oxide nucleation.^24^

Mahaffey et al.^29^ studied the behavior of HR-120 in pure sCO_2_ and at atmospheric pressure with ppm additions of O_2_ and CO. The study showed that this alloy forms Cr_2_O_3_ as the base oxide layer, but diffusion of Mn and Fe occurs to form spinels at the outer oxide layer. The presence of CO increased the carbon deposition rate at the oxide–substrate interface.^29^

A recent study by Ilinich et al.^30^ investigated the behavior of Inconel 740H, Inconel 625, Haynes HR-120, and 316L stainless steel to address inconsistencies in data pertaining to tests conducted in an sCO_2_ environment. The samples were held in sCO_2_ at 20 MPa and 700 °C for a total of 1500 h and were removed for mass gain measurements and characterization after 500, 1000, and 1500 h. The results of the study indicated that 740H developed the most protective oxide layer, composed primarily of Cr_2_O_3_ oxide with an underlying thin layer of Al_2_O_3_. The results of the study also indicated the need for an extended study to capture a more complete oxidation behavior of the alloys.^30^

Although numerous studies have examined oxidation and carburization of high-performance alloys in sCO_2_, the literature contains inconsistencies arising from CO_2_ purity, flow regimes, pressure, and test system design, and most standardized data sets are limited to ≤ 1500 h. As a result, the long-term evolution of protective oxides (e.g., Cr_2_O_3_), porosity/densification, and the coupled progression of carburization and Cr-depletion at 700 °C/20 MPa remain insufficiently characterized across key alloys (740H, 625, HR-120, 316L).

To address this gap, we extend the harmonized methodology of Ilinich et al.^30^ to 4800 h in sCO_2_ at 700 °C and 20 MPa, using the same alloy set. The experimental methodology is described in Section 2, including exposure conditions and characterization techniques (SEM-EDX and XRD). We perform time-resolved mass gain and microstructural analyses to quantify oxide scale development and Cr-depletion behavior and to assess substrate degradation beneath the oxide scale. While carburization is recognized as a potentially important degradation mechanism in sCO_2_ environments, its presence is evaluated qualitatively based on SEM/EDX and bulk XRD observations rather than through dedicated carburization diagnostics. We further adopt the total affected depth, defined here as the combined thickness of the oxide scale and the Cr-depleted region, as a design-relevant metric to compare alloys and provide mechanistic insights into duration-dependent degradation. These results yield a long-duration, standardized data set for materials selection and lifetime modeling in sCO_2_ Brayton systems.

The objectives are to (i) quantify long-term oxidation and associated microstructural degradation, (ii) characterize oxide scale evolution and Cr depletion beneath the scale, and (iii) assess the implications of these degradation modes for materials selection in sCO_2_ Brayton systems.

## 2 Experimental Methods

### 2.1 Sample Preparation

Four high-performance alloys, Inconel 740H, Inconel 625, Haynes HR-120, and 316L stainless steel (hereinafter referred to as 740H, 625, HR-120, and 316L, respectively), were subjected to prolonged exposure to an sCO_2_ environment in this work. Their nominal compositions are listed in Table 1.

**Table 1:** Nominal Compositions of the Commercially Available 740H, 625, HR-120, 316L

	Composition (wt %)																
Alloy	Ni	Fe	Cr	Co	Mo	Nb	Al	Ti	C	B	Mn	Si	S	Cu	P	W	N
740H	Bal	<3.0	24.5	20	0.1	1.5	1.35	1.35	0.03	<0.006	<1.0	0.15	<0.03	<0.5	0.03	-	-
625	Bal	<5.0	21.5	<1.0	9	3.65	<0.4	<0.4	<0.1	-	<0.5	<0.5	<0.015	-	<0.015	-	-
HR-120	37	Bal	25	<3.0	<2.5	0.7	0.1	-	0.05	0.004	0.7	0.6	-	-	-	<2.5	0.20
316L	12	Bal	17	-	2.5	-	-	-	0.03	-	2	0.75	0.03	-	0.045	-	0.10

Six samples of each alloy were machined into circular disks with a 12.7 mm diameter and a thickness between 1.3 mm and 2.7 mm (thickness varied for each alloy) and polished to 600# grit. The samples were cleaned and degreased using deionized water, ammonium hydroxide-based solution, propanol, and acetone. Following the sample preparation, the initial dimensions and weights of each sample were measured using a digital caliper (King Canada/KM-106) with an accuracy of 0.01 mm and an analytical balance (Schuler Scientific/MA-T-5) with an accuracy of 1.6 μg. The samples were stored inside a desiccator prior to the commencement of tests to avoid excess atmospheric oxidation. Six samples of each alloy were exposed to an sCO_2_ environment; five were removed, weighed, and characterized as part of the study, while the remaining samples were returned to the desiccator until the prolonged exposure testing was initiated.

### 2.2 Corrosion Testing and Sample Characterization

The samples were tested in a bench-scale high-pressure corrosion test rig, jointly developed by CanmetENERGY-Ottawa (CE-O) and Carleton University (CU). A detailed description and visual overview of the test rig can be found in previous studies.^30,31^ Continuing the test campaign initiated by Ilinich et al.,^30^ one sample of each alloy was exposed to industrial-grade CO_2_ (99.99% purity, supplied by Messer Group) in a supercritical state at 700 °C and 20 MPa for a total of 4808 h of exposure (4800 h nominally). The total exposure duration of ∼4800 h represents a continuation of the test campaign established previously^30^ and provides a meaningful window into long-term oxidation trends under sCO_2_.^13,16,20^ While this duration does not constitute a true lifetime assessment, it is sufficiently long to capture the mechanistic transitions that typically precede long-term degradation, namely chromia instability, Fe-rich oxide development, and depletion-zone evolution. Thus, the present results allow evaluation of the alloys’ long-term protective behavior within the scope of a bench-scale study, while recognizing that multi-year or component-scale testing would be required for full lifetime qualification. After 2000, 2500, 3000, 3500, and 4800 h, the test system was cooled down, depressurized, and the mass of each sample was recorded. The samples were characterized after the final exposure interval (4800 h). Note that the procedure followed for the characterization of the pre-exposure samples and after 1500 h of exposure is described in Ilinich et al.^30^

The samples were characterized using scanning electron microscopy (SEM), energy-dispersive X-ray spectroscopy (EDX), and X-ray diffraction (XRD) techniques. SEM and EDX analyses were performed using a Philips XL30 SFEG high-resolution scanning electron microscope by means of secondary electron (SE) and backscattered electron (BSE) methods. Samples used for surface analysis did not require additional preparation. For cross-sectional examination, the samples were cleaned and coated with a 40 nm layer of gold using an Edwards S150B sputter coater. The samples were then electroplated with copper using a fully saturated CuSO_4_ solution, a voltage of 0.1 V, a current of 150 mA, and an exposure time of 10 min. The plated samples were cold-mounted in an epoxy mixture and sectioned using a diamond blade. The sectioned samples were then polished using the following progressive polishing technique: sanding using 320-grit SiC paper, followed by polishing with 9 μm and then with 3 μm diamond abrasive, finishing with polishing using a 0.05 μm colloidal silica suspension. Between each polishing stage, the samples were cleaned in a Struers Lavamin cleaning unit, and the final cleaning step took place in an ultrasonic bath with a diluted soap solution. EDX was used to identify the elemental composition of the detected phases. It is worth mentioning that the sample preparation technique in this work differs from that used in the short-term exposure study presented earlier.^30^ This is an improved technique to preserve the oxide layer from separation during cross-sectional examination. XRD analysis using a Bruker D8 was applied to investigate the crystallography of the samples over a 2θ range of 20–90° at a scanning rate of 2.4°/min. The intensity of the peaks was examined using Jade9 software, which was equipped with the International Center for Diffraction Data (ICDD) database.

## 3 Results

Figure 1 displays the photographs of the samples at the start of the test and after exposure to sCO_2_ for 1500, 3000, and 4800 h. The results of the 1500 h exposure are presented in detail in the previous study,^30^ and the images are shown here for a visual representation of the oxidation progression. After 3000 h, the two Inconel samples (740H and 625) transitioned to a smoother surface finish, with a dull shine in appearance. 740H displayed a uniform surface colorization, whereas some dark spots were visible on the surface of 625. The top half of the HR-120 sample attained a brown tint. 316L showed signs of pitting on the surface facing the inlet of the sample boat and spallation of material from the sides of the sample. After 4800 h of exposure, 740H became lighter in color but maintained a dull shine. Similarly, 625 remained dull with dark spots on the surface. 316L showed spallation along the specimen edges and, most notably, on the front face of the sample, exposing a brown surface underneath. The surface oxide layer on HR-120 also began to show early signs of spallation, characterized by surface roughening.

**Figure 1:**
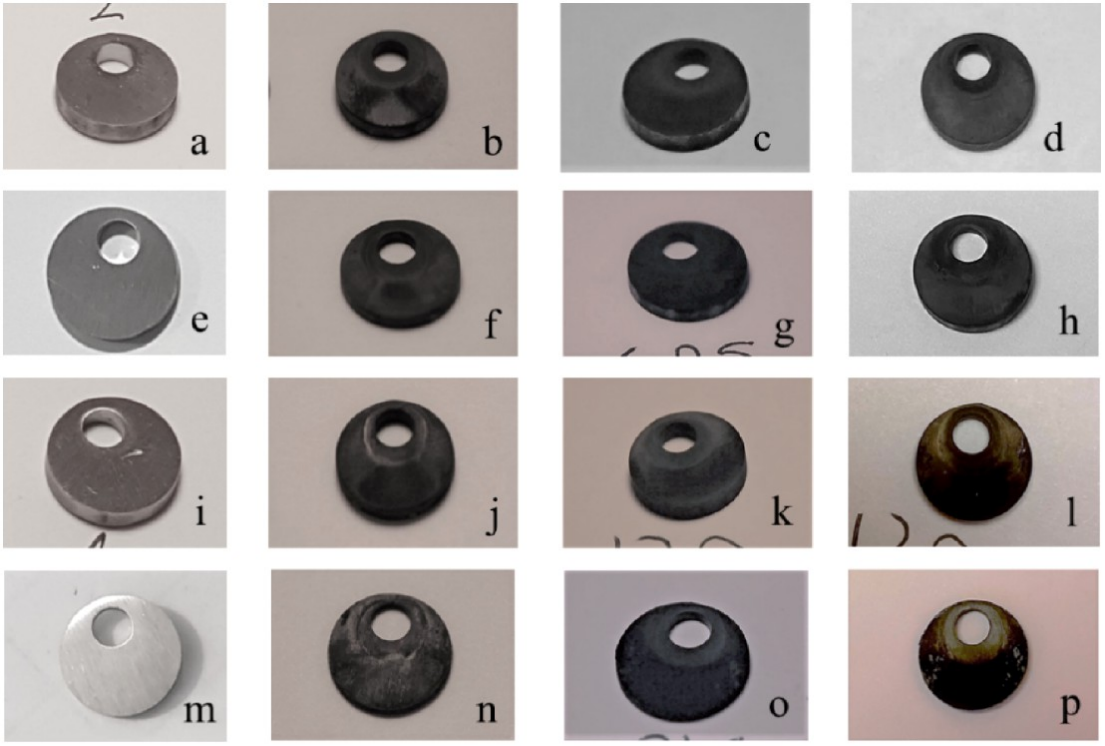
Visual appearance of the alloy samples exposed to sCO_2_ environment at 700 °C at 200 bar at 0 h (as received), 1500, 3000, and 4800 h exposure intervals (a-d) 740H samples; (e-h) 625 samples; (i-l) HR-120 samples; (m-p) 316L samples.

Figure 2 presents the mass changes of the four alloys across eight time intervals from 0 to 4800 h. For alloys 740H and 625, the mass-gain curves follow a predominantly parabolic trend up to approximately 2000 h, consistent with diffusion-controlled growth of a protective Cr_2_O_3_-based scale. Beyond ∼2000 h, a slight deviation from ideal parabolic behavior is observed, and the mass gain begins to increase more linearly. Given the limited number of data points at the longest exposure times, this late-stage behavior should be regarded as preliminary, and longer-duration exposures are currently ongoing to determine whether this trend persists or evolves with continued exposure. Additionally, given the limited magnitude of this deviation and the relatively small number of long-duration data points, this shift is attributed to an apparent departure from fully protective behavior. By the end of the test campaign, the two alloys, 740H and 625, increased in mass by 0.40 mg/cm^2^ and 0.31 mg/cm^2^, respectively.

**Figure 2:**
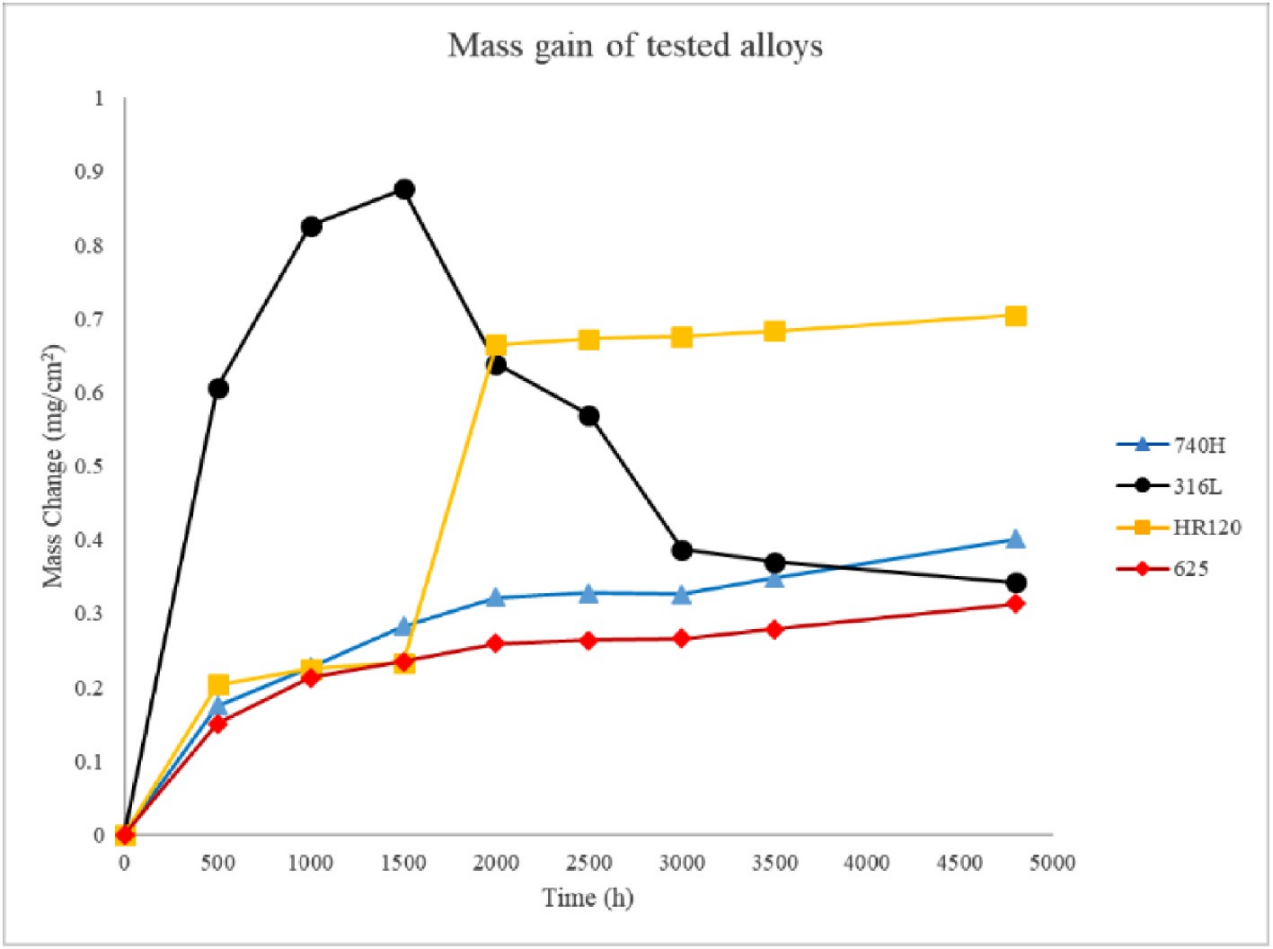
Mass change of the four alloys tested at 700 °C and 200 bar in an sCO_2_ environment for up to 4800 h.

HR-120 displayed a noteworthy change in oxidation behavior after 1500 h, denoted by a significant increase in mass gain between 1500 and 2000 h. Following 2000 h, the oxidation pattern appears to stabilize, as evidenced by Figure 2. 316L stainless steel shows a parabolic oxidation rate up to 1500 h, followed by a sudden mass loss after the 1500 h interval. Based upon the pattern of mass gain followed by mass loss, it is expected that the mass loss is due to spallation of the oxide layer; this was confirmed with visual examination of the surface. The rate of mass loss decreases beyond 2000 h of exposure.

Figure 3 exhibits the surface oxide growth for the four alloys and its progression from the start of testing (0 h, Figure 3a,d,g,j) to 1500 h (Figure 3b,e,h,k) and 4800 h (Figure 3c,f,i,l). As expected, the oxide growth increased significantly with the time of exposure for all alloys, which is consistent with the mass gain noted previously in Figure 2.

**Figure 3:**
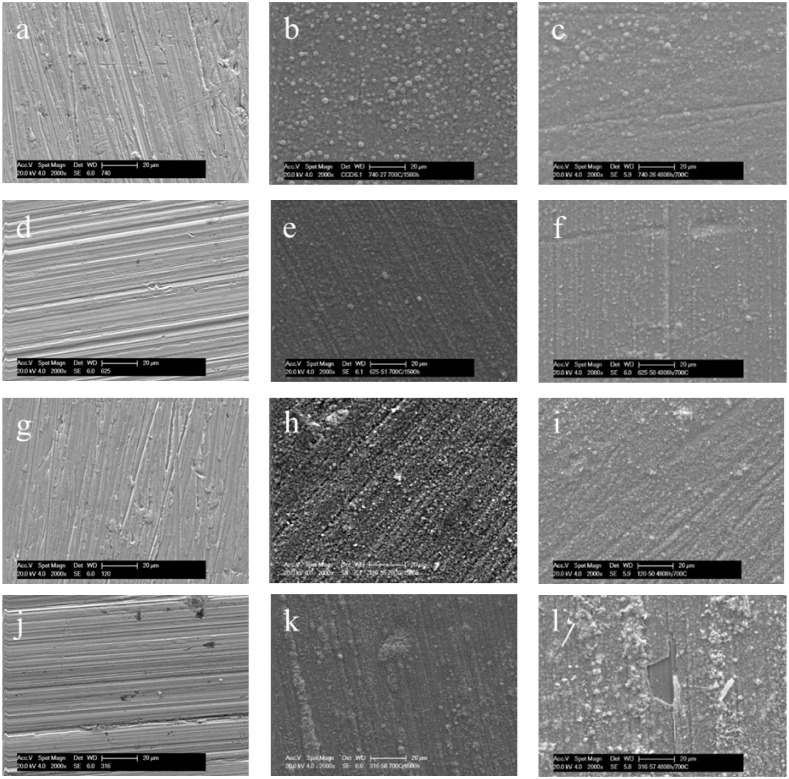
Surface oxide SEM imaging of the alloy samples exposed to the sCO_2_ environment at 700 °C at 200 bar at 0 h (as received), 1500 and 4800 h exposure intervals (a-c) 740H samples; (d-f) 625 samples; (g-i) HR-120 samples; (j-l) 316L samples.

Figure 3a,b,c shows the progression of surface oxide growth over the alloy 740H substrate at the three time intervals. It is evident that the base oxide layer increased in thickness and consumed the individual areas of increased oxide growth from 1500 to 4800 h. Additionally, it can be noted that the SEM images show amalgamation of the spherical oxides between 1500 and 4800 h (Figure 3b,c). At the higher magnification, it was observed that the shape of the elongated needlelike oxides transitioned to become shorter and rounded. In addition, the number of the individual oxide precipitates greatly reduced by the end of 4800 h of exposure (Figure 4a,b). The EDX area scans (Figure 5) indicated that the base oxide layer throughout the exposure is primarily composed of Cr-rich oxides. The oxide mounds on the surface of the alloy showed a high level of Cr content and a slight increase in Mn and Ti compared to the alloy composition. The spot scans (Figure 6) of high-aspect-ratio oxides also displayed high Cr content and some increase in Mn. These results were later confirmed by XRD analysis, which showed prominent peaks corresponding to Cr_2_O_3_, Al_2_O_3_, NiCr_2_O_4_, and MnCr_2_O_4_ (Figure 10a). When compared against the 1500 h results, sharpening of the MnCr_2_O_4_ peaks was noted, indicating an increase in their formation.^30^

**Figure 4:**
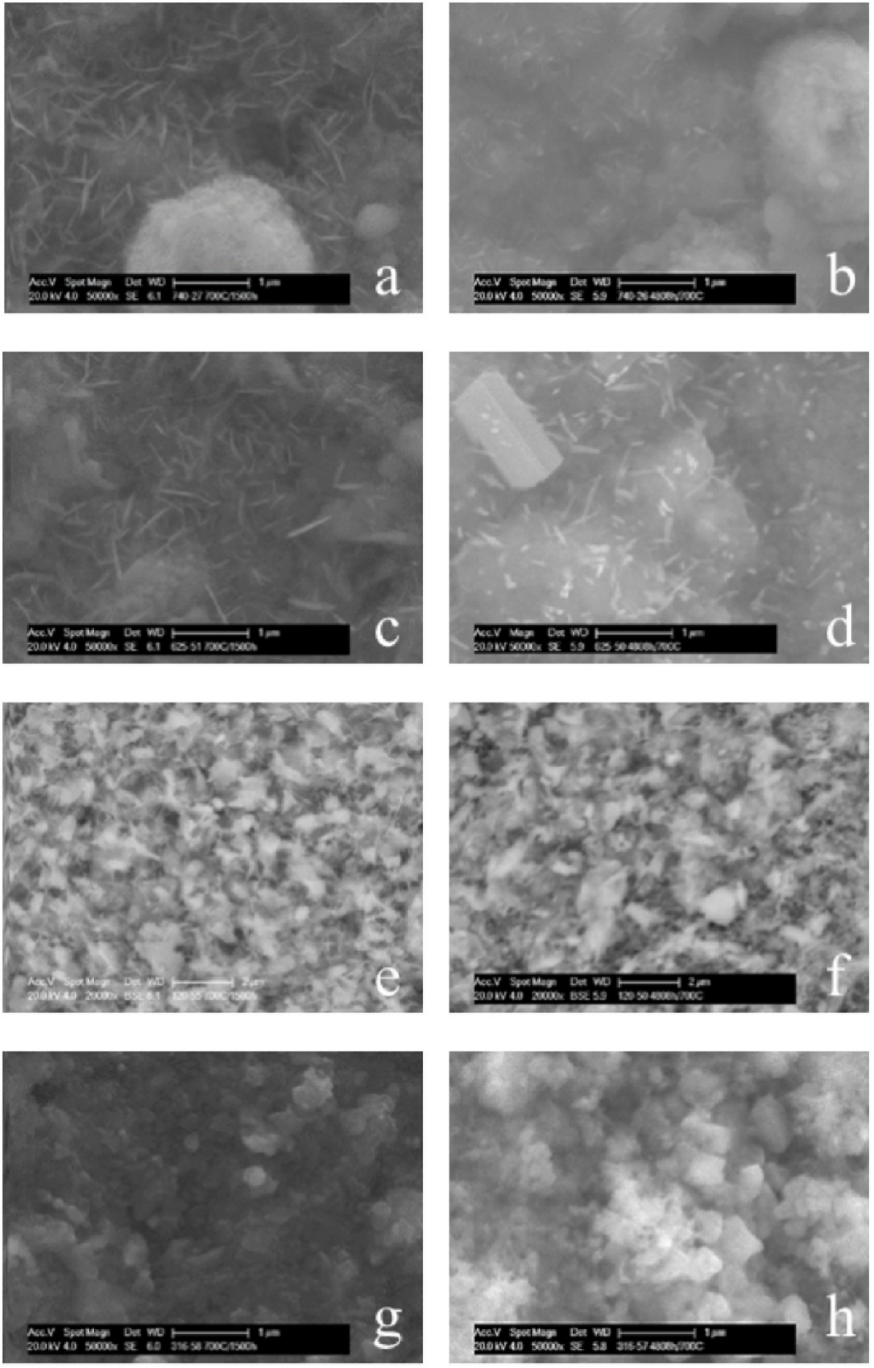
Surface oxide morphology of the sample coupons at higher magnification at 1500 and 4800 h exposure intervals (a-b) 740H; (c-d) 625; (e-f) HR-120; (g-h) 316L.

**Figure 5:**
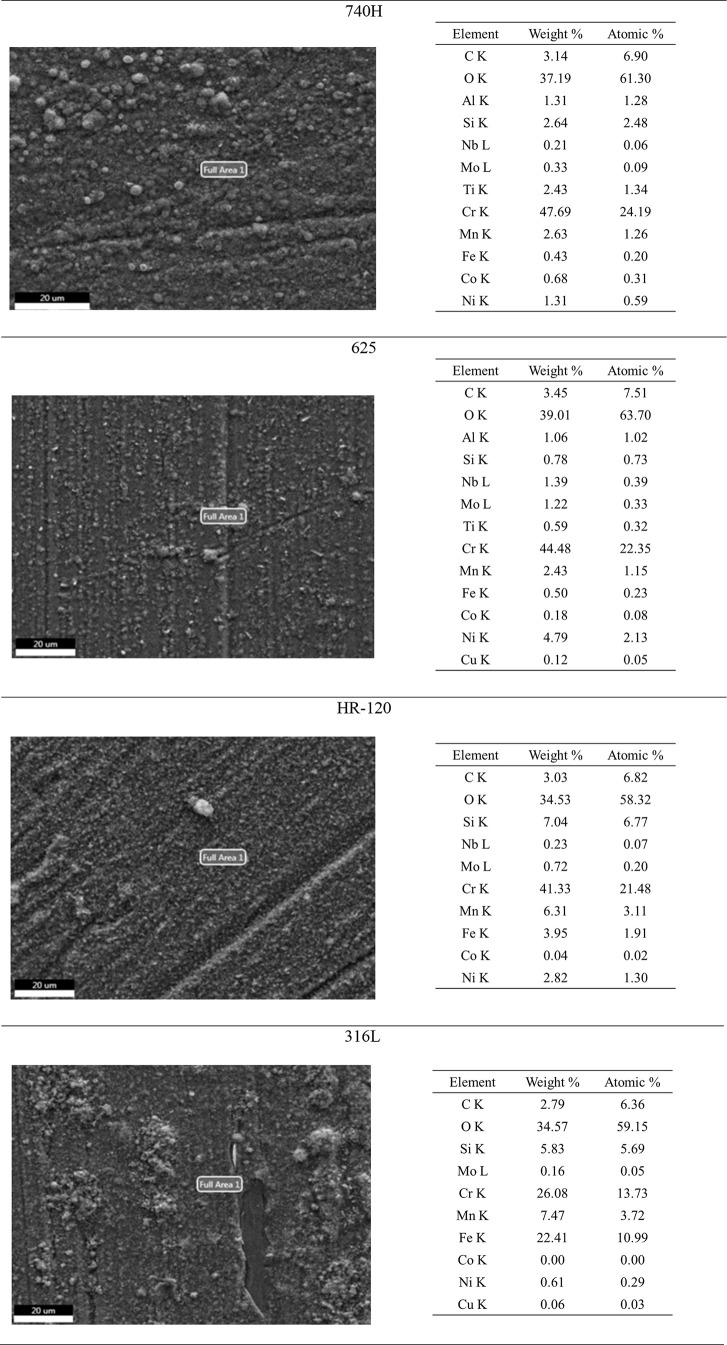
EDX area scans after 4800 h of exposure to 700 °C, 20 MPa sCO_2_ showing the predominant elemental compositions of the surface oxides.

**Figure 6:**
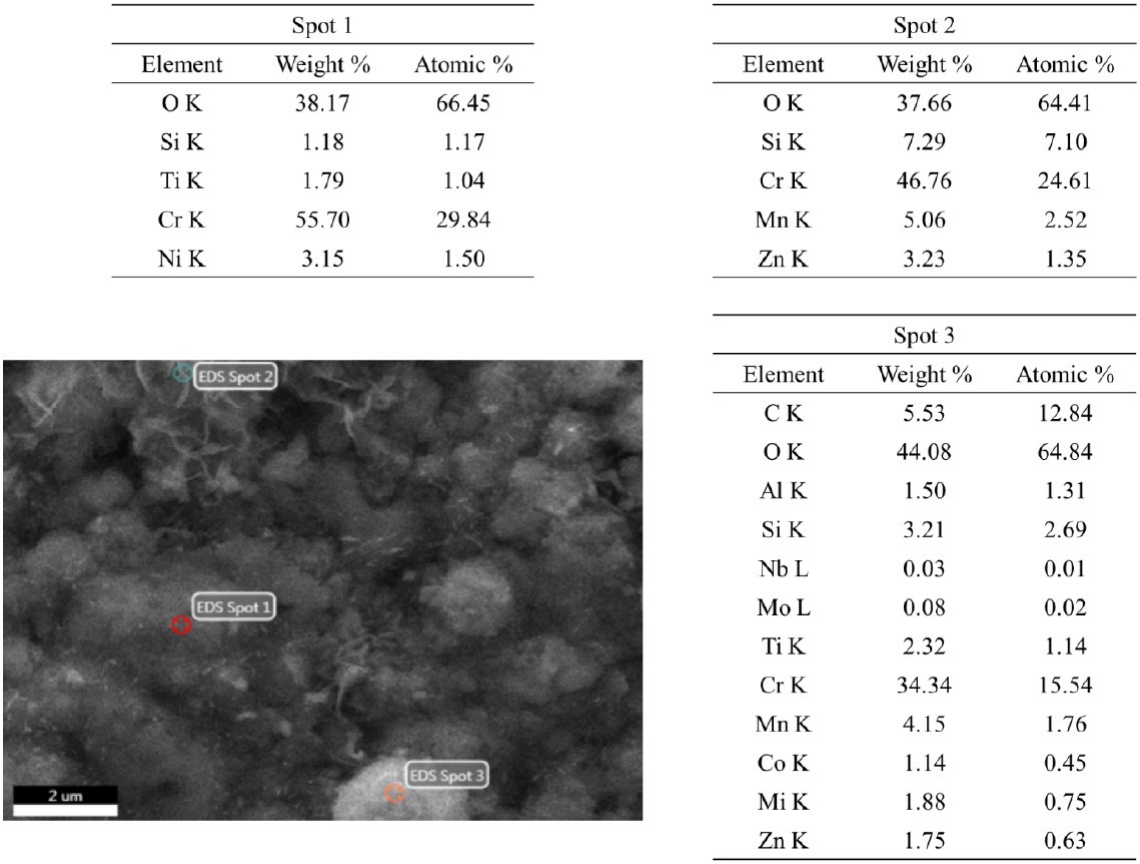
EDX spot scans of Inconel 740H following 4800 h of exposure to 700 °C, 20 MPa sCO_2_ with spot 2 and spot 3 predominantly showing Cr-based oxides with the presence of Al, Mn, and Mo and increased Si content when compared to spot 1.

The surface oxide of alloy 625 appears to be more uniform compared to that of alloy 740H, with fewer areas of increased oxide growth visible at lower magnification (Figure 3d,e,f). At higher magnifications, similar to those of 740H, one can see small oxide particles within the base layer. The length and shape of these particles changed with 1500–4800 h exposure, becoming shorter and less sharp (Figure 4c,d). The EDX area and spot scans for this alloy indicated a Cr-rich base layer, whereas the EDX spot scans (Figure 7) of areas of increased oxide formation showed an additional presence of Al, Mo, and Nb. The XRD scan confirmed the presence of Cr_2_O_3_ oxide on the surface, accompanied by MnCrO_3_, NiCr_2_O_4_, MnCr_2_O_4_, and Ni_3_(Mo,Nb), as shown in Figure 6. At 1500 h, chromia was undeniably the primary oxide; however, after 4800 h, the XRD scan showed a significant spike in the intensity of MnCr_2_O_4_ oxides, indicating an increase in formation.

**Figure 7:**
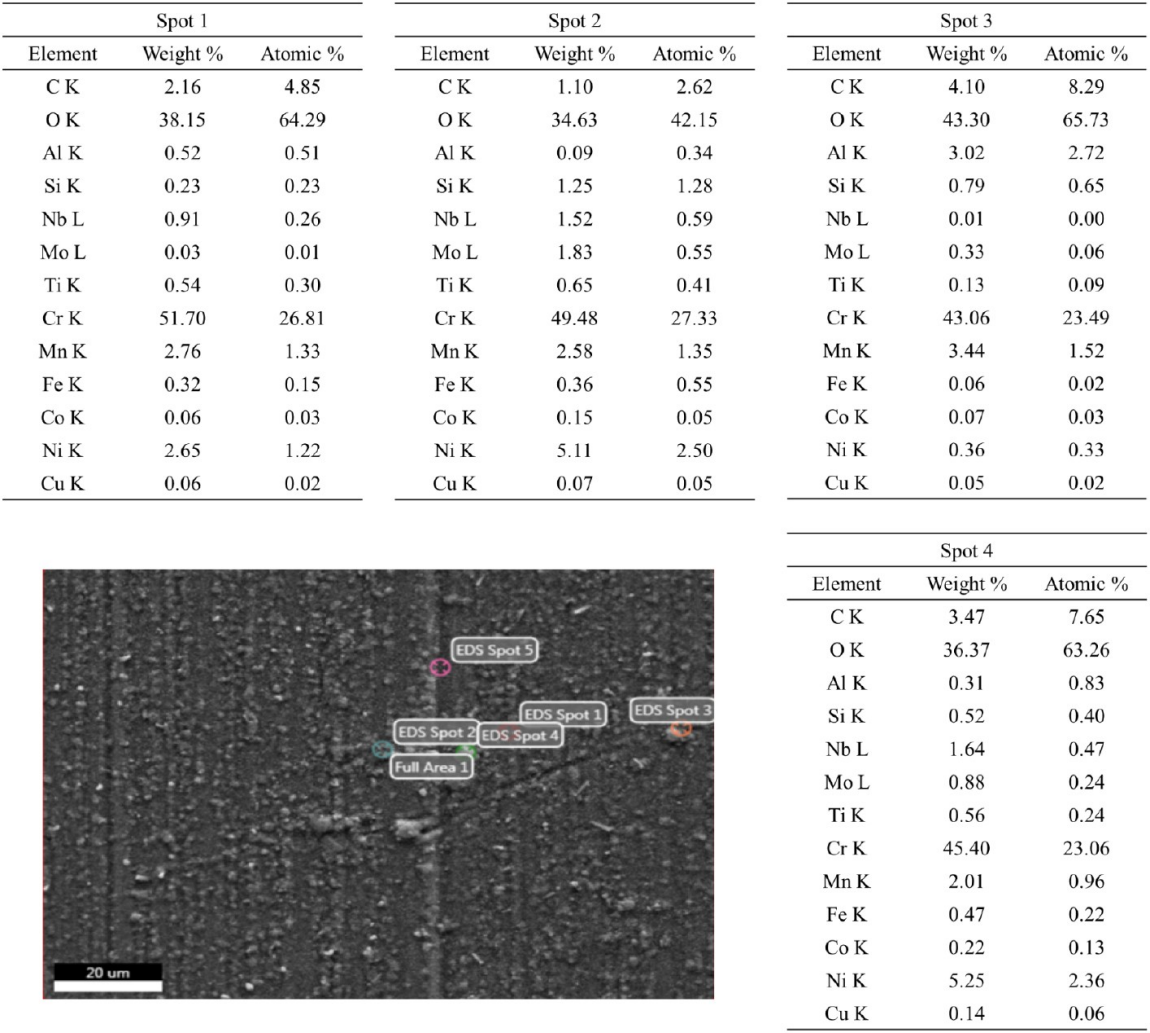
EDX spot scans of Inconel 625 following 4800 h of exposure to 700 °C, 20 MPa sCO_2_ with spot 1 showing increased Mo and Nb content when compared to spots 2, 3, and 4.

Hastelloy HR-120 exhibited a dual oxide morphology composed of granular and needlelike oxides (Figure 3g-i). At the higher magnification level, it can be noted that the size of the granular oxides did not change with the increasing length of exposure, but the number of needlelike oxide particles decreased (Figure 4e,f). EDX area scans (Figure 8) indicated an increased content of Cr and Mn in the base oxide layer. The spot scans of regions of greater oxide growth illustrated an increase in the Mo, Mn, and Si contents. The XRD scans confirmed that the main oxide is Cr_2_O_3_ with MnCr_2_O_4_ spinels (Figure 10c). Similar to the two alloys described above, it can be noted from the XRD scans that past the 1500 h of exposure to sCO_2_, the presence of MnCr_2_O_4_ oxide considerably increases.^30^

**Figure 8:**
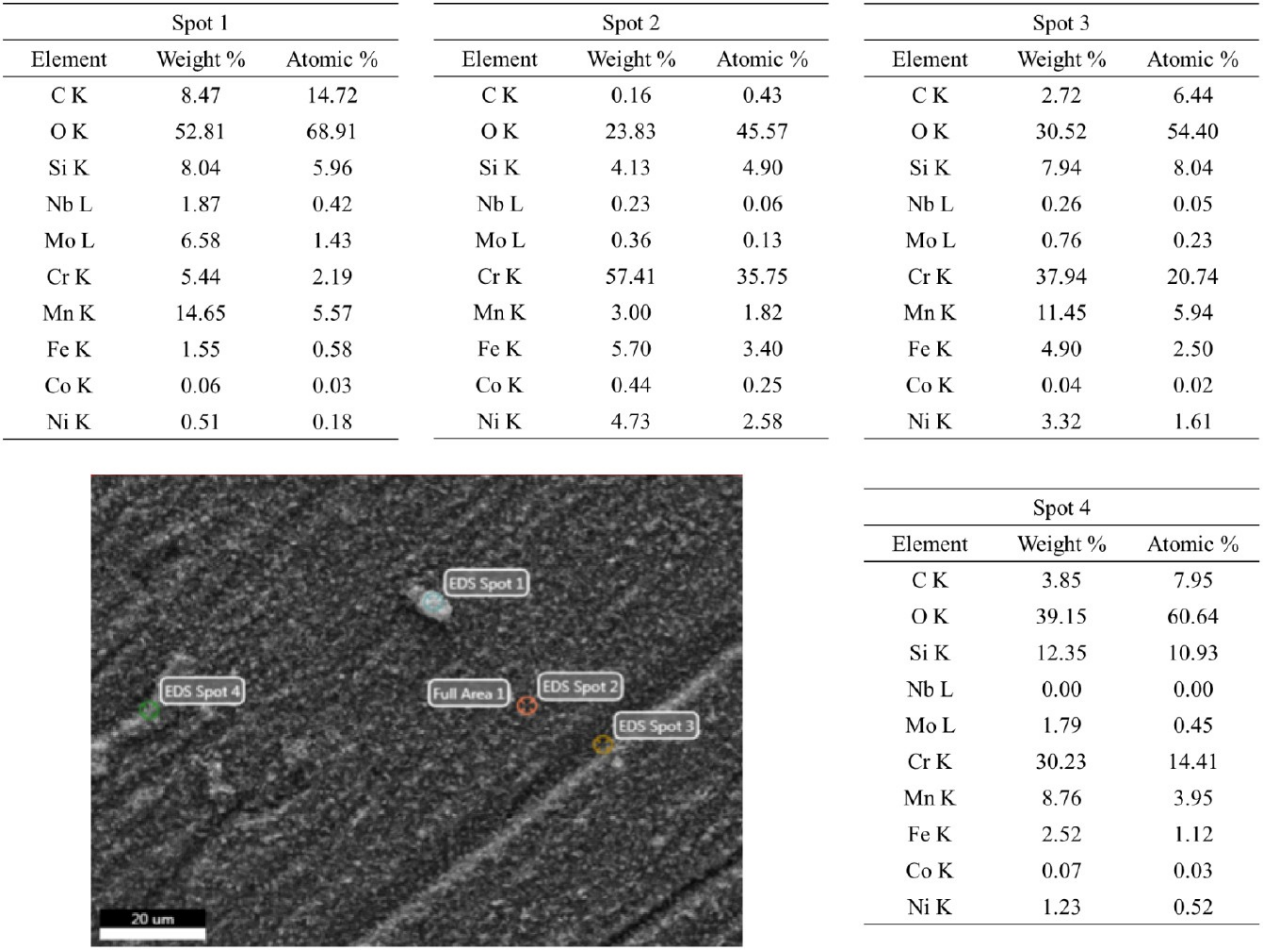
EDX spot scans of HR-120 following 4800 h of exposure to 700 °C, 20 MPa sCO_2_ with spot 1 showing increased Mo and Nb content when compared to spots 2, 3, and 4.

Figure 3j,k,l shows the surface oxidation of 316L stainless steel, where the alloy exhibits a thick base oxide layer with many areas of increased oxidation. On Figure 3l, one can also see significant spallation, consistent with the visual examination of the sample coupon and the mass change data in Figure 2. At higher magnification, the sample coupon exhibits granular oxide morphology at 1500 and at 4800 h (Figure 4g,h). The EDX area scans (Figure 5) identified the presence of Cr- and Fe-based oxides, whereas spot scans (Figure 9) showed that the areas of excessive oxide growth were mainly Fe-rich, whereas the surrounding areas were Cr-based. The spot scans of the surface beneath the spallation detected oxygen, in addition to high levels of Cr and Fe. This elemental analysis indicates that the spallation occurred during the sample’s exposure to sCO_2_ and a new layer of oxide began to form. The XRD analysis (Figure 10d) confirmed these observations, showing that after 4800 h, Cr_2_O_3_ and Fe_2_O_3_ remained the primary oxides, with high levels of Fe_3_O_4_ and MnFe_2_O_4_. Some FeMnO_3_ peaks were also observed on the XRD scan. Compared against the XRD scan of the sample after 1500 h exposure, there was not a significant change in the oxide type.^30^

**Figure 9:**
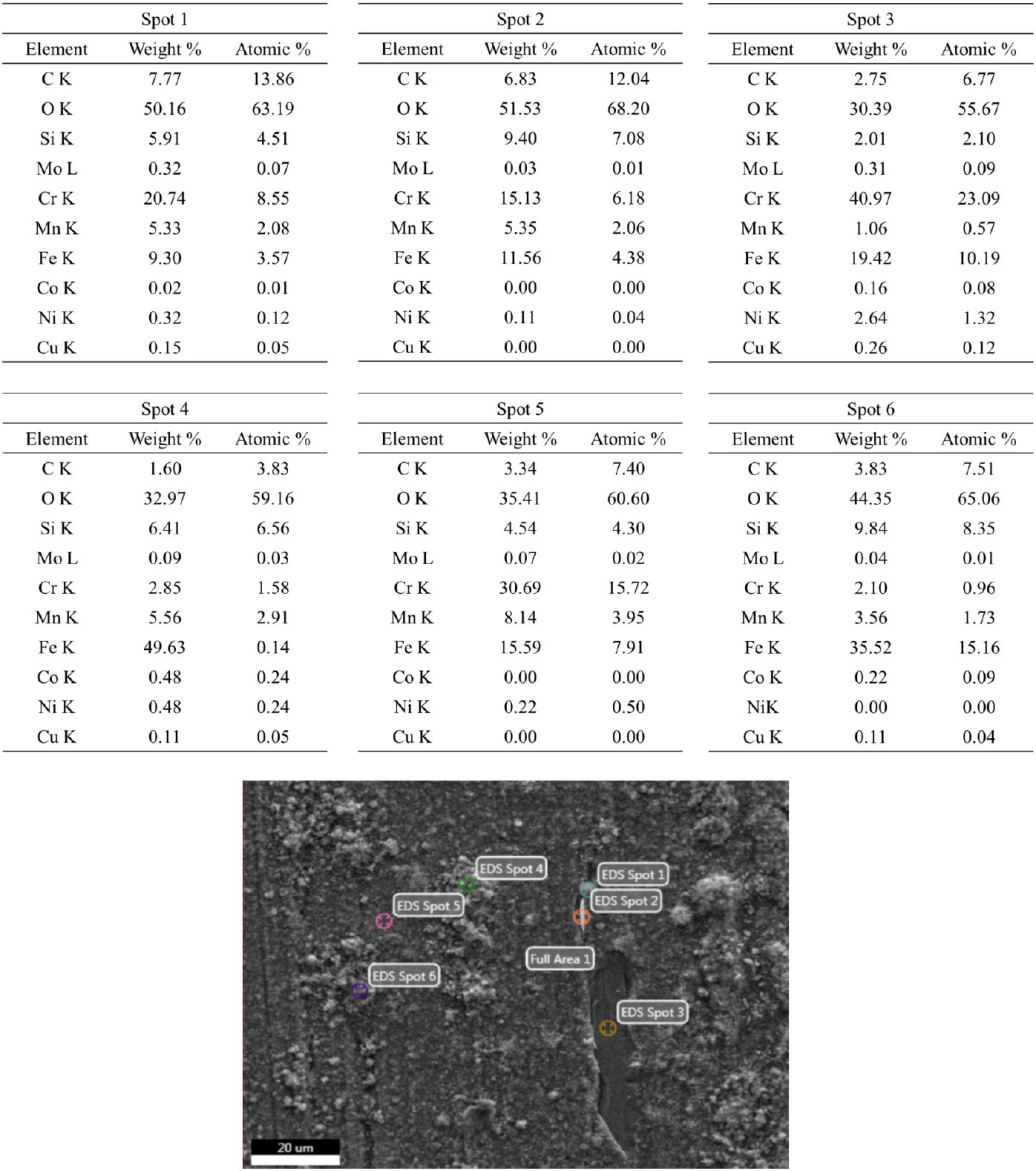
EDX spot scans of 316L following 4800 h of exposure to 700 °C, 20 MPa sCO_2_ with spot 3 showing exposed substrate and spots 4 and 6 highlighting areas of increased oxide growth with greater Fe content than is seen in the baseline oxide (spot 5).

**Figure 10:**
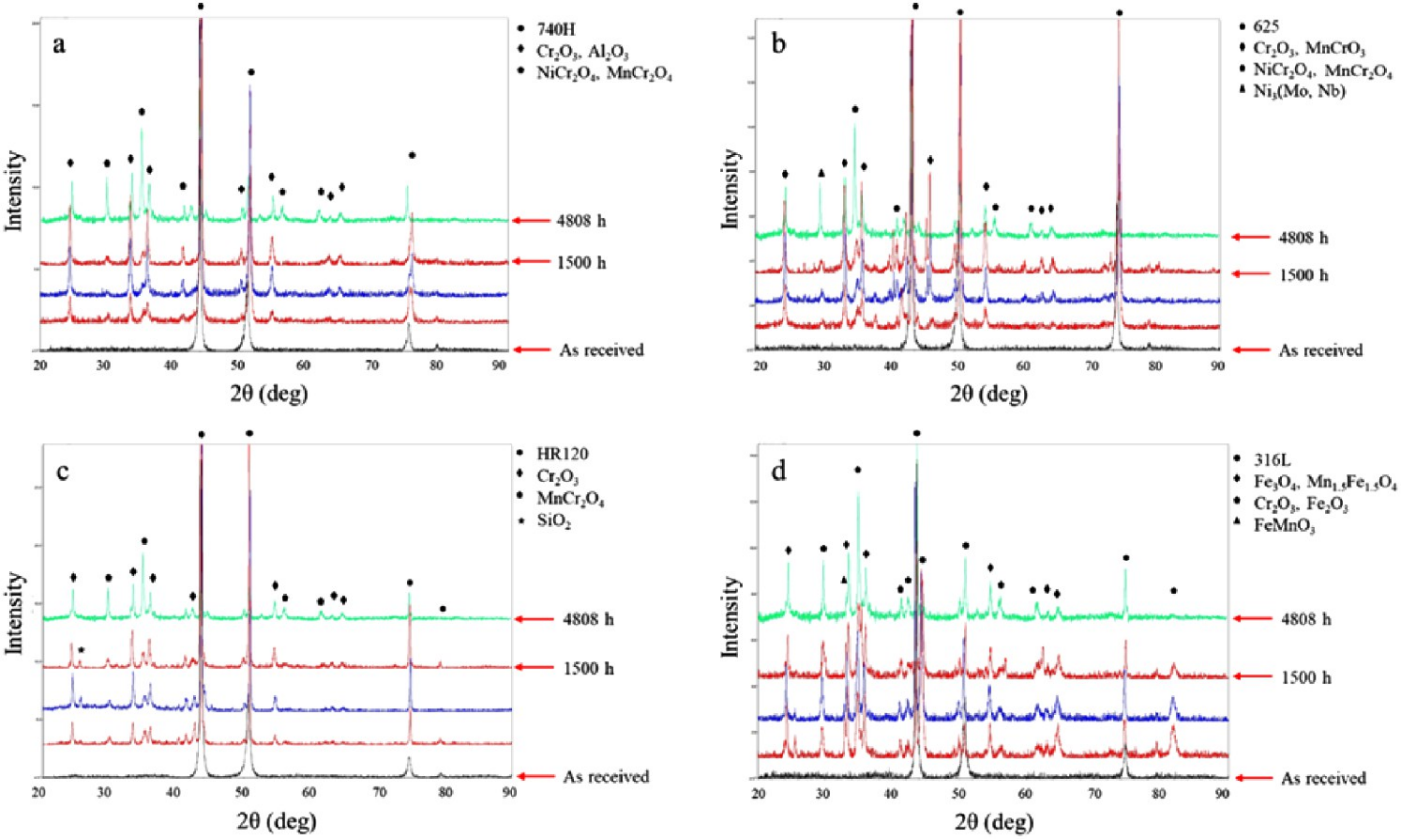
XRD scans after 4800 h exposure to sCO_2_ at 700 °C, 20 MPa of (a) Inconel 740H; (b) Inconel 625; (c) Hastelloy HR-120; (d) 316L stainless steel.

Figure 11a,b shows the cross-sectional examination of 740H under 5000× and 25000× magnifications. The images reveal a mostly uniform oxide layer and significant void content near the substrate surface. The previous study of the oxidation behavior up to 1500 h reported the formation of intergranular voids near the oxide–substrate interface.^30^ After 4800 h of exposure to sCO_2_, these voids are being filled with Al-rich oxides. These oxides are shown as dark spots along grain boundaries in Figure 11b. The average thickness of the oxide layer after 4800 h was measured to be 2.46 ± 0.23 μm, an increase from 1.81 ± 0.06 μm after 1500 h. The increase in oxide thickness corresponds to the increase in mass measurements. Similarly, the Cr-depletion zone was measured at 5.66 ± 0.18 μm after 4800 h, increased from 3.99 ± 0.17 μm after the first 1500 h. The EDX line scans (Figure 12a) show a high content of Ni at the gas–oxide interface, transitioning into a Ni–Cr-based outer oxide. A thick layer of Cr_2_O_3_ oxide forms the primary oxide scale, with a thin independent discontinuous layer of Al_2_O_3_ detected beneath.

**Figure 11:**
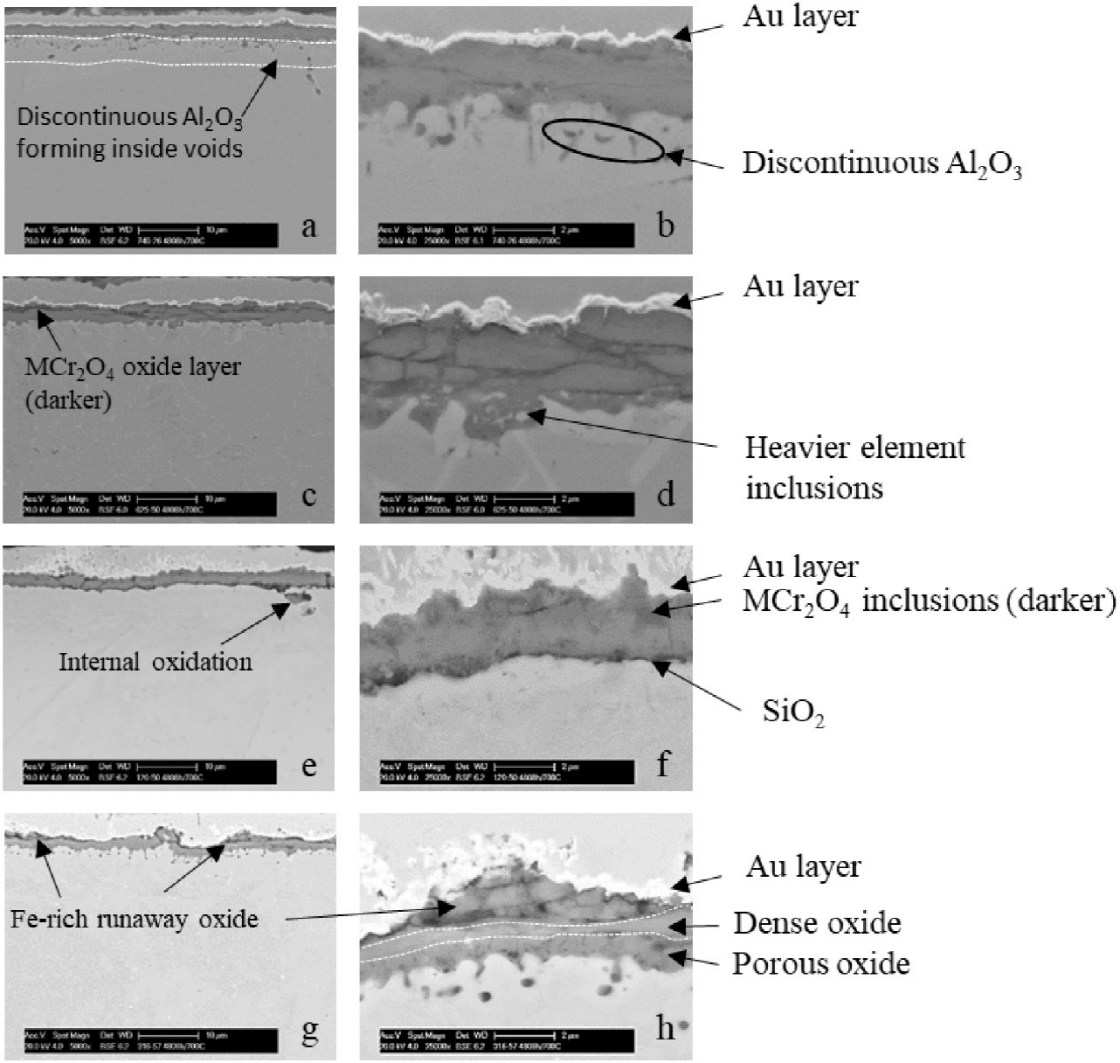
Cross-sectional SEM after the exposure for 4800 h at 5000× and 25000× magnification for (a-b) Inconel 740H; (c-d) Inconel 625; (e-f) Hastelloy HR-120; (g-h) 316L stainless steel.

**Figure 12:**
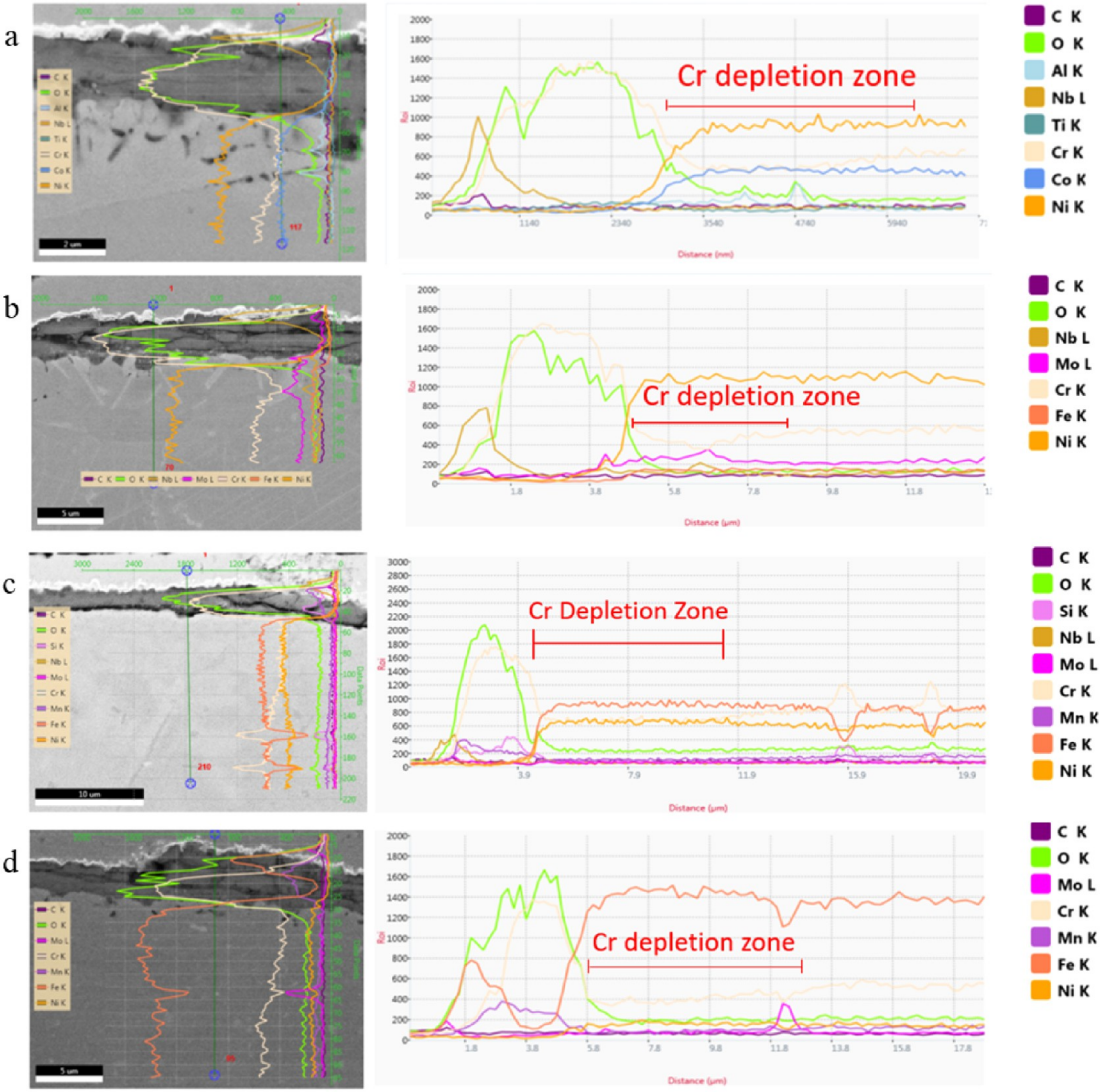
EDX line scans of oxide and substrate after the 4800 h exposure for (a) Inconel 740H; (b) Inconel 625; (c) Hastelloy HR-120; (d) 316L stainless steel.

The cross-sectional examination of alloy 625 is shown in Figure 11c,d. In Figure 11d, one can see high-porosity regions, which exhibit lower adhesion to the substrate. Toward the substrate, the oxide layer incorporates heavier elements, indicated by the lighter inclusions within the oxide layer in Figure 11d. Furthermore, the oxide–substrate interface contains an increased content of heavy-element precipitates as can be observed by lighter areas in Figure 6c,d. The surface oxide thickness increased from 1.09 ± 0.16 μm to 3.26 ± 0.09 μm from 1500 to 4800 h, respectively. Unlike alloy 740H, alloy 625 maintained almost the same thickness of the Cr-depletion layer, 4.44 ± 0.28 and 4.47 ± 0.16 μm for the two time intervals, respectively. Within the inner oxide, two distinct morphologies are observed: (i) dense regions, characterized by compact oxide with minimal internal voiding, and (ii) porous regions, where microscale voids and channels are present due to vacancy condensation during outward metal-ion diffusion. As labeled in Figure 11b,d,f,h, the dark bands and pockets correspond to porous regions; EDX line scans (Figure 12) acquired across these zones show comparable Cr-rich chemistry to adjacent dense oxide but a reduced O signal, consistent with their higher void fraction. The heavy elements noted in the BSE scans were identified to be primarily Nb. Similar to 740H, the EDX scan revealed an increase of Ni at the gas–oxide interface, followed by a small section of Ni–Cr-based oxide. Underneath the oxide scale and coinciding with the Cr-depletion zone, the scans display an increase in Mo content.

Cross-sectional images of alloy HR-120 are shown in Figure 11e,f. Figure 11e shows an area of internal oxide formed in a void within the substrate, a development not seen in the previous study of the exposure to sCO_2_ up to 1500 h.^30^ The BSE scanning mode reveals a darker oxide near the oxide–substrate interface, signaling the presence of lighter elements. The EDX line scan (Figure 12c) shows elevated Ni at the outer oxide layer, similar to 740H and 625, however, in a much smaller quantity. The main oxide formed during the oxidation of HR-120 is Cr_2_O_3_ containing Mn-based spinels, consistent with the XRD scan in Figure 10c. The Mn content is the highest at the furthest distance from the substrate, within the oxide scale, tapering off with increasing depth of oxide. A noteworthy observation is the presence of Si-containing oxide near the oxide–substrate interface. This is the likely cause for the dark layer in Figure 11. The average oxide thickness at 1500 h was recorded to be 1.94 ± 0.51 μm, whereas at 4800 h, it was measured at 2.70 ± 0.22 μm. It is important to note that the oxide measurement at 1500 h may not represent the true oxide thickness due to oxide loss during sample preparation. The Cr-depletion zone was measured at 5.44 ± 0.38 and 7.13 ± 0.58 μm for the two intervals, respectively.

Figure 11g,h shows the oxide propagation in 316L stainless steel. The duplex morphology of the oxide layer continued from the previous study.^30^ In localized regions where the protective chromia layer deteriorated, the oxide developed a three-layered structure consisting of (i) an inner Cr-rich oxide adjacent to the substrate, (ii) a Fe-rich spinel layer, and (iii) an outer Fe oxide layer. This multilayered morphology, which is typical of 316 under high-temperature CO_2_ exposure when chromia cannot be sustained, was observed in thicker oxide regions identified along the cross-section (Figure 11). The outer Fe oxide layer showed signs of incipient spallation, consistent with commonly reported breakaway behavior. To assess spatial variability, six oxide thickness measurements across the width of the sample were carried out (full data sets provided in the Supporting Information). Measurements were not taken at isolated spots of potential runaway oxidation but rather at representative regions along the cross-section, and the SEM field shown in Figure 11h is representative of the overall morphology observed in this data set. The intermediate oxide layer appears more coherent. Spallation observed for 316 is more plausibly linked to the formation of a thicker Fe-rich outer oxide following chromia breakdown, which generates higher growth stresses as the duplex Fe oxide develops. Additional cross-sectional SEM images were not obtained capable of capturing these thicker regions directly; however, the comparatively greater mass gain for 316 suggests localized development of such Fe-rich layers under the exposure conditions. Another noteworthy feature is the substrate layer directly beneath the oxide. Figure 11g displays the lack of an even substrate surface, leading to the formation of stresses within the oxide layer during heating–cooling cycles and fast-tracking the spallation of the oxide scale.

The increased population of voids immediately beneath the oxide–metal interface (as shown in Figure 11g) indicates enhanced vacancy accumulation during prolonged exposure. Such voids can form through vacancy injection at the oxide–metal interface associated with outward cation diffusion during oxide growth, where flux imbalance between metal cations and oxygen leads to vacancy supersaturation in the substrate. In addition, continued consumption and depletion of reactive alloying elements (e.g., Cr) required to sustain oxide growth can further contribute to void formation and stabilization. Consequently, the observed increase in void density likely reflects the combined influence of outward-growing oxide kinetics and progressive alloy depletion, rather than a single dominant mechanism. The cross-sectional examination of the 4800 h sample did not reveal any regions where the oxide scale had detached or was locally missing due to spallation as had been seen in previous work.^30^ However, the layer of discontinuous oxide in Figure 11g is indicative of the cross-sectional morphology of the Fe_3_O_4_ excess oxidation. The EDX line scans (Figure 12d) confirm the layered nature of the 316L oxide with the base layer primarily consisting of Cr and CrMn- based oxides, while the outer layer oxide showed to be primarily composed of Fe-based oxide. Significantly, the low cohesion zone at the surface is primarily Fe-based oxide whereas the low cohesion oxide near the substrate appears to be primarily Cr-based. The oxide thickness was difficult to measure due to oxide layer spallation and uneven oxide formations. The Cr-depletion zone was measured to be approximately 5.11 ± 0.65 and 6.37 ± 0.35 μm after 1500 and 4800 h, respectively.

## 4 Discussion

The assessment of the usability of materials in industrial applications relies heavily on the performance of long-term oxidation studies. As can be seen from Figure 3, the oxidation kinetics can change with the extended exposure, which highlights the importance of extended exposure studies. For the first 2000 h, alloys 740H and 625 follow the parabolic oxidation law according to eq 1:^32^

1 $$W^{2}=K_{p}t$$

where *W* is the weight gain (mg·cm^–2^), *K*_p_ is the parabolic diffusion rate constant (mg^2^·cm^–4^·s^–1^), and *t* is the exposure time (seconds).

After the 2000 h, it can be noticed that the oxidation becomes more linear, according to eq 2:^33^

2 $$W=K_{l}t$$

where *W* is the weight gain (mg·cm^–2^), *K*_l_ is the linear diffusion rate constant (mg·cm^–2^·s^–1^), and *t* is the exposure time (seconds). Linear rate constants were extracted from the later-stage mass-gain data to provide a comparative description of oxidation behavior after deviation from parabolic kinetics, rather than to imply the establishment of a true steady-state linear oxidation regime. It should be emphasized that the apparent deviation from parabolic kinetics observed at longer exposure times reflects the current extent of the data set rather than a conclusively established steady-state oxidation regime. Continued exposure to longer durations is ongoing and will be required to determine whether the observed behavior stabilizes, accelerates, or transitions further with time.

Table 2 summarizes the *K*_p_ and *K*_l_ for alloys 740H and 625 and provides an *R* ^2^ value to justify the relationship (1.15 × 10^–8^ mg^2^·cm^–4^·s^–1^ and 1.14 × 10^–8^ mg·cm^–2^·s^–1^ and 6.44 × 10^–9^ mg^2^·cm^–4^·s^–1^ and 7.25 × 10^–9^ mg·cm^–2^·s^–1^, respectively). These numbers fall in line with previously reported oxidation rates for the two Inconel alloys.^34^ For both alloys, the oxidation rate exhibited an initial parabolic behavior, followed by a deviation toward a more linear trend after the 2000 h interval as evidenced by Figure 2. The parabolic oxidation rate is indicative of a protective oxide layer, formed through ionic diffusion, characterized by high adhesion to the substrate.^35^ This behavior is commonly observed in nickel and chromium oxides, including Cr_2_O_3_. On the other hand, the linear oxidation rate is observed for porous oxides that do not offer an effective barrier to oxygen penetration. These oxides are thus susceptible to cracking and spallation.^35^ This process has also been shown to facilitate the diffusion of the metal ions through the protective Cr- and Al-rich layers, as observed in several studies.^24,25,35–37^ This transition can be attributed to several factors, including (i) Cr depletion below critical level which is needed to sustain the Cr_2_O_3_ oxide layer, i.e., reaching a critical oxide thickness, (ii) formation of less protective spinel oxides, and (iii) thermal cycling, i.e., shutdown and startup cycles between intervals.^16,20,27,35,38,39^ Indeed, in this study, the composition of the oxide layer transitions from being primarily composed of Cr_2_O_3_ to containing high amounts of MCr_2_O_3_, where M represents Mn, Ni, Nb as per the XRD analysis (Figure 5a,b). In addition, slight Cr depletion beneath the oxide–substrate interface can be observed from line scans (Figure 12a,b); however, it is unlikely that the level of depletion is indicative of critical Cr_2_O_3_ thickness. Furthermore, cracking within the oxide layers was observed in both sets of materials (Figure 11a,b,c,d). If these cracks formed as a result of thermal cycling, they could facilitate the diffusion of the metal ions, such as Ni, Mn, and Nb, through the protective Cr- and Al-rich layers to form less protective oxides, as observed in this study (Figure 10a,b) and in the literature.^24,25,29,37^ Additionally, alloy 740H formed a substantial number of intergranular voids underneath the oxide layer, further providing routes for diffusion and the formation of internal oxidation. This observation was also noted by Jiang et al. during the evaluation of 740H at temperatures above 1000 °C for use in ultrasupercritical power plants. The authors explain the pore formation with diffusion of Cr and Ti ions.^27^

**Table 2:** Oxidation Rates of the Four Alloys

Alloy	*K*_p,Interval 1_ (mg^2^·cm^–4^·s^–1^)	*R* ^2^_Interval 1_	*K*_l,Interval 2_ (mg·cm^–2^·s^–1^)	*R* ^2^_Interval 2_
740H	1.15 × 10^–8^	0.96	1.14 × 10^–8^	1.00
625	6.44 × 10^–9^	0.92	7.25 × 10^–9^	1.00
HR-120	3.54 × 10^–9^	0.94	3.93 × 10^–9^	0.99
316L	1.11 × 10^–7^	0.90	-	-

On the other hand, Hastelloy HR-120 shows clear evidence of the transition in oxidation behavior after 1500 h. For the first 1500 h, the data fits best under the parabolic law with *K*_p_ of 3.54 × 10^–9^ mg^2^·cm^–4^·s^–1^, followed by a breakaway behavior resulting in a subsequent more linear trend (*K*_l_ of 3.93 × 10^–9^ mg·cm^–2^·s^–1^). These results are summarized in Table 2 and confirm the behavior that is commonly observed in low-Cr or high-Fe alloys.^23,24^ The oxidation rate reported here for the initial 1500 h period is higher than values in the literature,^34^ which may be attributed to the CO_2_ composition used during testing. Industrial-grade CO_2_ (99.99% purity) contains minor impurities, including N_2_ (<70 ppm), O_2_ (<20 ppm), and moisture (<10 ppm), that can influence the oxidation behavior,^17,40^ whereas laboratory-grade CO_2_ typically reaches 99.999% purity. After 1500 h, exposure to sCO_2_ led to the development of voids beneath the oxide scale,^30^ which promoted the formation of internal oxides thereafter, as seen in Figure 11e. As evidenced in the line scan presented in Figure 12c, the presence of an extended Cr-depletion zone, compared to the Inconel samples, implies that the oxidation kinetics of the alloy increasingly rely on the transport of Ni and Mn to form unprotective spinels. Ni enrichment at the outer oxide layer of HR-120 likely reflects the evolution of the oxide scale under the sustained Cr depletion, contributing to increased scale complexity and internal stress development rather than the formation of a protective barrier. This chemistry shift, together with partial pore infill and scale densification, can increase mass uptake without a commensurate increase in apparent thickness within a given field of view. While the representative cross-section in Figure 11 shows a thickness comparable to the Inconel alloys, the full cross-section of the samples was surveyed, and six oxide-thickness locations and five Cr-depletion depths were quantified (Supporting Information). No runaway nodules were captured at those positions. Localized thickening below our sampling pitch, combined with thermal-cycle-induced cracking between intervals, could also contribute to the observed mass increase. In HR-120, for example, the presence of an underlying SiO_2_ layer and the observed mass-gain acceleration may be consistent with degradation pathways that also permit carbon ingress if continuous chromia protection is lost. Targeted carburization diagnostics were not performed in this study, and resolving this conclusively remains a priority for future work. Additionally, the presence of an underlying SiO_2_ layer combined with the observed mass-gain acceleration may indicate degradation pathways that also permit carbon ingress if continuous chromia protection is lost. However, targeted carburization diagnostics were not performed in this study and resolving this conclusively remains a priority for future work.

316L stainless steel showed very poor oxidation resistance after 1500 h, consistent with similar studies.^24–26,41^ The initial parabolic oxidation rate, *K*_p_ = 1.11 × 10^–7^ mg^2^·cm^–4^·s^–1^, was the highest among all alloys tested, indicating that 316L was the fastest oxidizing material in the present study. Although some disagreements exist among the oxidation rates reported in the literature, the value obtained here is within the expected range for 316L under similar high-temperature conditions.^34^ Severe spallation was first observed between 1500 and 2000 h of exposure, corresponding to the sharp drop in mass-gain measurements. This behavior is consistent with continued formation of Fe_3_O_4_ oxide and related spinels, which are known to be highly porous, poorly adherent, and unprotective. Unlike chromia, Fe_3_O_4_ readily develops through-channels, facilitating rapid outward diffusion of Fe cations and inward transport of oxidizing species. This transport mechanism accelerates internal oxidation and sustains continued oxide thickening without establishing a diffusion barrier.^24,25^

As Fe migrates outward to form the growing Fe-oxide scale, vacancy supersaturation develops at the metal–oxide interface; these vacancies condense into voids, which may be subsequently occupied by Fe_*x*_Cr_3_-_*x*_O_4_ spinels according to the “available-space model”,^24^ as can be observed by the overlapping Cr, Fe, and O line scans in Figure 12d. Due to the extent of oxide delamination and material loss, it is not possible to reliably extract long-term oxidation kinetics for 316L beyond 1500 h, and therefore, no linear rate constant is reported for this alloy in Table 2.

Overall, the formation of a multilayered oxide scale is generally undesirable because maintaining adhesion requires close compatibility in thermal expansion coefficients between both the substrate and the individual oxide layers.^35^ When multiple oxide types are present, mismatches in thermal expansion behavior create internal stresses that promote cracking or delamination, particularly under thermal cycling associated with the heating and cooling of the system. This microstructural instability can also increase the susceptibility to secondary degradation processes.

At high magnifications, alloys 740H and 625 both exhibited the development of needlelike oxide particles in the oxide matrix leading up to 1500 h as reported in the previous study.^30^ However, during the examination of 4800 h results, it was noted that the shape of these oxides changed, becoming shortened and more rounded. HR-120 and 316L did not reveal a significant change in morphology. 740H continued developing intergranular voids underneath the oxide layer. Such voids, when interconnected, may serve as short-circuit pathways for the outward diffusion of metal cations during continued sCO_2_ exposure, thereby contributing to accelerated local oxide scale growth.^24,25,27,29,37^ Furthermore, discontinuous Al_2_O_3_ oxides continued growing beneath the primary Cr-based oxide. A continuous Al_2_O_3_ oxide layer would positively impact high-temperature oxidation resistance by acting as a diffusion-limiting barrier; however, such layers typically form only at sufficiently high Al contents (>2–6 at.%) and/or elevated temperatures (>950 °C).^42,43^ The present study does not satisfy these conditions, and thus the Al_2_O_3_ observed beneath the chromia scale in 740H forms as discontinuous internal precipitates rather than as a continuous protective sublayer. Consequently, these Al_2_O_3_ features are unlikely to directly reduce oxidation kinetics, even at extended exposure times. Instead, the superior long-term oxide performance of 740H observed here is attributed primarily to the continuity and structural stability of the chromia scale. In Inconel 625, primary Cr_2_O_3_ oxide formation continued; a secondary oxide layer enriched in heavier elements was formed near the substrate, and a region of Ni_3_Mo precipitates was noted, which may influence the substrate surface roughness. Haynes HR-120 showed signs of internal oxidation after 4800 h. In 316L, oxides continued to form in a duplex morphology, and a triplex structure was developing in areas of severe oxidation, accompanied by significant void content in the substrate. Lastly, Inconel 740H displayed void development along grain boundaries, and horizontal cracks in the oxide layer were observed after 4800 h. Although alloy 625 exhibited the lowest mass gain, the localized cracking within its chromia layer and the presence of secondary heavy-element oxides and Ni_3_Mo precipitates suggest a scale that may be less stable under thermal or mechanical gradients than the primarily Cr_2_O_3_ oxide structure observed in 740H.

Cross-sectional examination of all four alloys revealed areas of increased porosity in the protective oxide layers, most prominently in 316L and 625. The substrate–oxide interface exhibited differing degrees of roughness for the four alloys, with HR-120 showing the least roughness and void content. Both 316L and 740H exhibited significant void content at the substrate surface and an increased surface roughness. The oxide–substrate interface roughness of 625 may be influenced by the presence of heavy-element inclusions, as seen in Figure 11. Despite the cracking and porosity within the oxide scales, only 316L exhibited clear spallation during the extended exposure. The Cr-depletion zone depth ranged from roughly 4.47 μm for alloy 625 to 7.13 μm for HR-120 and did not correlate with the interface roughness. Samples were not subjected to externally applied stresses beyond thermal expansion and contraction; imposed stresses or thermal cycling may reduce the oxide adherence, particularly for alloys with rough or defect-prone interfaces. In this comparison, the thinner Cr-depletion zone and the presence of a predominantly chromia-based oxide scale in 740H suggest more efficient utilization of Cr in maintaining a structurally stable oxide scale. The oxide morphologies observed during the surface scans did not exhibit significant changes between 1500 h^30^ and 4800 h. In alloys HR-120, 625, and 740H, where needlelike oxides were present after 1500 h, a change in shape and quantity was observed with longer exposure.

The evolution toward a denser and more compact outer morphology suggests a progression toward a more adherent and mechanically stable oxide scale.^40^ This morphological evolution aligns with the reduction in Cr_2_O_3_ peak intensities and the corresponding increase in the MCr_2_O_4_ spinel intensities observed in the XRD patterns. Among the alloys, 740H exhibited the most uniform transition toward a stabilized oxide morphology, consistent with the formation of a continuous and structurally stable chromia-based scale, whereas 625 and HR-120 exhibited more heterogeneous transformations that may reflect ongoing internal stress evolution or secondary-phase formation during extended exposure.

Oxide integrity is an important characteristic of alloys used in high-temperature applications. The formation of a continuous, well-adhered, and dense oxide layer is critical in mitigating runaway oxidation. Once formed, this layer continues to grow and requires a sufficient supply of oxidizing elements.^44^ Spallation occurs when a critical thickness is reached, triggered by accumulated thermal stresses. The formation of Cr_2_O_3_ results in a more gastight oxide layer than the porous Fe_3_O_4_, preventing CO molecules from penetrating and oxidizing the underlying metal.^24^ The formation of an inner Al_2_O_3_ oxide layer provides even greater protection by limiting the diffusion of heavier metals. SiO_2_ similarly enhances high-temperature oxidation resistance by inhibiting Ni transport.^40^ The Cr-depletion zone is also a critical indicator of long-term oxidation behavior; as the oxide grows, it consumes Cr, Al, and Si near the surface. Excess depletion reduces the mechanical strength, promotes crack initiation, and accelerates fatigue failure.^45^ Minimizing depletion requires stable oxide adhesion to avoid excessive consumption of the metal ions. When the Cr content in the substrate drops below the critical level needed to sustain a protective chromia scale, the alloy transitions to forming less-protective oxides such as Fe_3_O_4_ or NiO. This loss of chromia stability can eventually lead to breakaway oxidation and may, under continued growth stresses or thermal cycling, contribute to spallation.^24^ Table 3 summarizes oxide thicknesses and depletion zone depths for all four alloys. Taken together, these metrics, oxide architecture, interfacial stability, and depletion-zone characteristics, provide a broader basis for evaluating oxide integrity than mass-gain measurements alone, explaining why 740H is interpreted as forming the most structural stability despite the lower mass gain observed for alloy 625.

**Table 3:** Oxide Scale and Cr-Depletion Zone Thicknesses for the Four Alloys after 4800 h of Exposure to sCO_2_

Alloy	Oxide thickness (μm)	Cr-depletion zone thickness (μm)
740H	2.46 ± 0.23	5.66 ± 0.18
625	3.26 ± 0.09	4.47 ± 0.16
HR-120	2.70 ± 0.22	7.13 ± 0.58
316L	1.93^a^ ± 0.26	6.37 ± 0.35

a The sample underwent severe spallation.

On the basis of these results, it is evident that HR-120 and 316L have the least amount of Cr available to continue building up the protective oxide layer. Once exhausted, metals such as Mn and Mo will continue diffusing to form the oxide scale until the onset of spallation. 625 has the thinnest region of Cr depletion, signaling the largest availability of protective oxide-building elements.

Carburization of materials exposed to sCO_2_ is an important aspect to be studied. Diffusion of carbon into the substrate will result in the formation of carbides, which can negatively impact mechanical properties. While several studies have reported the absence of increased bulk carbon within the substrate, thin C-rich layers at or near the oxide–substrate interface have also been reported. In this study, carburization was not directly characterized beyond SEM/EDS and bulk XRD, which are not diagnostic for detecting subsurface carbon ingress or fine carbide precipitation beneath the Cr-depletion zone. The EDX line scans for all four alloys did not show an unequivocal increase in the level of carbon within the substrate or directly beneath the oxide layer. For 740H, a small C spike coinciding with increased Ni at the gas–oxide interface was observed; area/spot EDX and XRD provided no evidence of carbide formation (Figure 5a), and we attribute the spike to Au/Cu coating interference, consistent with a prior study.^46^

For Ni-based alloys (740H and 625) that largely sustained protective chromia, these observations do not contradict the view that carburization is less likely; however, they do not prove its absence. Given the porous or locally degraded oxide observed for 316L and HR-120, carburization remains plausible for the Fe-based alloys and cannot be ruled out by the present methods. Targeted carburization diagnostics were not performed in this study, and resolving this conclusively remains a priority for future work.

While sCO_2_ Brayton cycles offer thermodynamic advantages, their economic viability depends on balancing material costs with performance benefits. High-performance alloys such as Inconel 740H and 625 are over 10 times more expensive than austenitic steels;^47^ however, their superior oxidation and carburization resistance can extend component life by several years, reducing replacement costs and downtime. Thus, strategically deploying these high-performance Ni-base alloys only in the sections exposed to the most severe operating conditions (e.g., ∼700 °C and ∼20 MPa) enables system developers to balance upfront material costs with long-term durability, ultimately improving the economic viability of sCO_2_ Brayton cycle technology.

Our findings on oxide integrity and Cr-depletion behavior provide design-relevant inputs for lifetime prediction models while highlighting the need for targeted carburization diagnostics in future work. For example, alloys with lower total affected depth under extended exposure can reduce maintenance frequency, improving the levelized cost of electricity (LCOE). Future work should integrate these degradation metrics into full techno-economic models to quantify life cycle savings and optimize material selection for commercial deployment.

## 5 Conclusion

Alloys 740H, 625, HR-120, and 316L were subjected to sCO_2_ at 700 °C and 20 MPa for an extended duration of 4800 h. Samples were examined through mass gain measurements and after exposure, using SEM in SE and BSE modes, EDX, and XRD to determine the oxide morphology, composition, and crystal structure. Both the surface of the samples and a cross-sectional area were examined. The following are the main conclusions of the study:

1 Inconel 740H and 625 exhibited the most predictable oxidation behavior by following the parabolic oxidation rate law up to 2000 h, then deviating toward a more linear relationship. 625 was measured to have the lowest mass gain up to 4800 h. Hastelloy HR-120 experienced a critical runaway oxidation point at 1500 h, significantly increasing the mass gain. 316L stainless steel experienced oxide layer failure in the form of spallation at 1500 h.
2 It is expected that 740H formed the most structurally stable oxide: a primarily Cr_2_O_3_ oxide layer, with underlying discontinuous Al_2_O_3_ oxides. While 625 also exhibited a primarily Cr_2_O_3_ oxide layer, it was supplemented with a secondary oxide layer enriched in heavier elements near the substrate (e.g., Ni_3_Mo). At longer exposures, the presence of intergranular voids may accelerate oxidation in 740H, while the presence of heavier elements may decrease the stability of the Cr-rich oxide in 625. 625 was noted to have the thinnest Cr-depletion zone, signaling the availability of metal ions for maintaining the oxide scale. HR-120 formed a fairly uniform oxide, with an underlying SiO_2_ layer. 316L formed the most porous oxide among the studied alloys.
3 On the basis of the methods employed here (SEM/EDS and bulk XRD), we did not observe diagnostic evidence of carburization; however, these techniques are not definitive for detecting subsurface carbon ingress or fine carbide precipitation. Consequently, carburization cannot be ruled out, particularly for Fe-based alloys (316L and HR-120), where compact chromia was not sustained. Future work will include targeted carburization assessments, including metallographic etching to reveal carbides.

Despite these findings, several challenges and limitations remain with respect to material performance and system-level feasibility. Laboratory autoclave exposures do not fully replicate plant environments, where thermal gradients, start–stop cycles, mechanical stresses, and high-velocity flow can accelerate oxide spallation, mass gain transients, and synergistic degradation mechanisms. Additionally, the economic viability of using high Ni/Cr superalloys for large-scale components must be weighed against material cost, fabrication constraints, and weldability considerations. Broader system-level issues, including heat-exchanger fouling, pressure drop evolution, and integration of dry-cooling technologies in CSP systems, also require evaluation to understand long-term operational behavior.

Future work should therefore focus on addressing these technical gaps to better assess the feasibility of deploying these alloys in commercial sCO_2_ systems. In this context, the ∼4800 h exposures reported here represent an intermediate stage of an ongoing long-duration testing campaign, with continued exposure currently in progress. Multifactor experiments incorporating thermal cycling, mechanical stress, and controlled impurity levels (e.g., H_2_O, CO, SO_*x*_, hydrocarbons) are necessary to capture realistic degradation modes. Impurity-driven effects are particularly important given that contaminants can arise from CO_2_ decomposition, seal interactions, or leakage, and CO_2_-containing flue gas from oxy-combustion represents another relevant service environment. Component-scale testing, including weldments, bends, and formed geometries, should explore localized oxidation and microstructural evolution under operating stresses. Long-duration flow-loop studies can further clarify erosion, deposit formation, and scale adherence in dynamic conditions. Finally, integrating material-degradation data into techno-economic frameworks will enable lifecycle cost assessment, inform alloy-selection strategies, and support the design of durable high-temperature sCO_2_ Brayton systems.

## Supplementary Material



## Notes

Additional characterization data for the four alloys are provided. Tables S1–S4 provide a summary of the oxide thickness and Cr-depletion zone depth for each alloy at different time intervals (PDF)
