# Supplementary material for: The Effects of Extended Exposure to Supercritical CO2 on Select High-Performance Alloys
Source: ACS Omega. 2026 Jun 2;11(23):33416–33. doi: 10.1021/acsomega.5c11290 (PMC13280682; doi:10.1021/acsomega.5c11290)
Supplement: Supplementary file 1 [file ao5c11290_si_001.pdf]

The Effects of Extended Exposure to Supercritical CO_2 on Select High-Performance Alloys

Margarita Ilinich [1], Taylor Robertson [2], Dongyi Seo[2], Kourosh Zanganeh [1], Holly Dole [1*], Hamid Radfarnia [1], Henry Saari [3], Ashkan Beigzadeh [1]

[1] Natural Resources Canada (NRCan), 1 Haanel Drive, Ottawa, Ontario, K1A 0E4, Canada
[2] National Research Council of Canada, 1200 Montreal Road, Ottawa, Ontario, K1A 0R6, Canada
[3] Carleton University, Department of Aerospace and Mechanical Engineering, 1125 Colonel By Drive, Ottawa, Ontario, K1S 5B6, Canada
*corresponding author: holly.dole@nrcan-rncan.gc.ca

Supporting Information

Table S1: Oxide thickness measurements and Cr depletion zone depth measurements of HR120

Sample	500 h	1000 h*	1500 h*		4808 h	
**Measurement**	Oxide thickness (µm)	Oxide thickness (µm)	Oxide thickness (µm)	Cr depletion zone depth (µm)	Oxide thickness (µm)	Cr depletion zone depth (µm)
**1**	0.76	0.59	0.92	4.63	2.39	8.71
**2**	0.36	0.97	3.63	6.01	1.97	6.83
**3**	1.20	1.03	2.22	6.63	3.04	6.43
**4**	0.75	0.85	0.57	5.14	2.47	5.52
**5**	1.01	0.59	3.14	4.78	3.49	8.15
**6**	1.94	1.37	1.18	-	2.82	-
**Average**	1.01	0.97	1.94	5.44	2.70	7.13
**Standard Deviation**	0.54	0.30	1.25	0.85	0.54	1.30
**Standard Error**	0.22	0.12	0.51	0.38	0.22	0.58

*Due to loss of oxide phase during sample preparation these measurements denote observed intact oxide but may not represent true oxide thicknesses

Table S2: Oxide thickness measurements and Cr depletion zone depth measurements of 316L

**Sample**	**500 h**	**1000 h***	**1500 h***		**4808 h**	
**Measurement**	Oxide thickness (μm)	Oxide thickness (μm)	Oxide thickness (μm)	Cr depletion zone depth (μm)	Oxide thickness (μm)	Cr depletion zone depth (μm)
**1**	1.36	2.57	1.65	5.58	1.15	6.27
**2**	0.89	3.82	1.48	7.27	2.37	5.50
**3**	1.19	2.33	1.88	3.39	1.53	7.57
**4**	1.31	2.20	1.70	4.34	1.40	6.54
**5**	1.35	3.78	1.95	4.95	2.71	5.97
**6**	1.60	2.67	1.09	-	2.42	-
**Average**	1.29	2.89	1.63	5.11	1.93	6.37
**Standard Deviation**	0.23	0.72	0.31	1.45	0.65	0.77
**Standard Error**	0.10	0.29	0.13	0.65	0.26	0.35

*Due to loss of oxide phase during sample preparation these measurements denote observed intact oxide but may not represent true oxide thicknesses

Table S3: Oxide thickness measurements and Cr depletion zone depth measurements of 625

**Sample**	**500 h**	**1000 h**	**1500 h**		**4808 h**	
**Measurement**	Oxide thickness (μm)	Oxide thickness (μm)	Oxide thickness (μm)	Cr depletion zone depth (μm)	Oxide thickness (μm)	Cr depletion zone depth (μm)
**1**	1.37	1.62	1.02	4.22	3.09	4.80
**2**	0.74	1.50	1.28	4.78	3.67	4.19
**3**	0.7	1.31	1.04	3.51	3.23	4.33
**4**	0.89	0.95	0.60	5.14	3.19	4.14
**5**	0.63	1.55	0.91	4.61	3.03	4.89
**6**	0.43	1.76	1.76	-	3.36	-
**Average**	0.82	1.45	1.09	4.44	3.26	4.47
**Standard Deviation**	0.32	0.29	0.39	0.62	0.23	0.35
**Standard Error**	0.13	0.12	0.16	0.28	0.09	0.16

Table S4: Oxide thickness measurements and Cr depletion zone depth measurements of 740H

Sample	500 h	1000 h	1500 h		4808 h	
**Measurement**	Oxide thickness (μm)	Oxide thickness (μm)	Oxide thickness (μm)	Cr depletion zone depth (μm)	Oxide thickness (μm)	Cr depletion zone depth (μm)
**1**	0.52	1.04	1.83	4.54	1.76	5.30
**2**	0.60	0.77	1.55	4.17	2.31	5.91
**3**	0.75	1.42	1.86	3.95	2.78	5.20
**4**	0.51	0.85	1.99	3.75	2.14	6.16
**5**	0.27	1.47	1.72	3.53	2.40	5.74
**6**	1.43	0.80	1.92	-	3.39	
**Average**	0.68	1.06	1.81	3.99	2.46	5.66
**Standard Deviation**	0.40	0.31	0.16	0.39	0.56	0.41
**Standard Error**	0.16	0.13	0.06	0.17	0.23	0.18
